# C2 Solitary Bone Plasmacytoma Curettage and Vertebral Augmentation in an 83-Year-Old Female: Case Report and Review of Surgical Treatment Approaches in the Spine

**DOI:** 10.1155/2017/5692402

**Published:** 2017-12-17

**Authors:** Matthew J. Yousif, Taha A. Faruqi, Rakesh Ramakrishnan, Nilesh M. Patel

**Affiliations:** ^1^Beaumont Hospital Farmington Hills, Farmington Hills, MI, USA; ^2^Beaumont Hospital Oakwood Dearborn, Dearborn, MI, USA

## Abstract

Surgically, solitary bone plasmacytoma (SBP) of the craniocervical junction (CCJ) is typically treated with cement augmentation and occipital-cervical stabilization (OCS). In the orthopedic spine literature, various surgical treatment options have been described for SBP, but only a few studies exist describing SBP of the CCJ with treatment involving cement augmentation alone. We report the case of an 83-year-old female found to have C2 SBP that was successfully treated with curettage and cement augmentation alone.

## 1. Introduction

Solitary bone plasmacytoma (SBP) is a localized plasma cell tumor accounting for 2–5% of plasma cell malignancies. The median age of SBP is 55. When compared to multiple myeloma, SBP occurs more frequently in younger patients and has a much better prognosis [1, 2]. SBP has a 2-3 times higher incidence in males compared to females, whereas multiple myeloma displays an equal gender incidence [3]. Patients typically present with severe neck pain with movement before the onset of neurologic symptoms [2, 4]. On computed tomography (CT), SBP usually presents as a large, well-demarcated, cyst-like lesion [1]. Criteria for diagnosis of SBP include a radiographic lytic lesion, monoclonal plasma cell infiltration on biopsy, histologically normal bone marrow aspirate, absent or low concentration of serum and urine protein on electrophoresis, and absence of anemia, hypercalcemia, or renal impairment [1, 5].

Plasma cell neoplasms of the craniocervical junction (CCJ) are rare. When they do occur, early recognition is important as they can lead to cervical spine instability and sudden death. They often respond to radiation therapy and, however, can also be managed surgically. Lesions of the cervical spine are typically operated on if there is evidence of spinal instability, algesia, or neurologic compromise [5]. Surgical treatment guidelines do not currently exist for plasma cell neoplasms of the CCJ [2, 5]. Surgically, SBP of the spine is treated based on location, degree of involvement of vertebrae, and preference of surgeon. Various treatment approaches discussed in the literature include vertebroplasty, occipitocervical stabilization (OCS), laminectomy, the use of intraoperative MRI, and two staged surgeries, amongst others [6–9].

Few cases of plasmacytoma of the cervical spine treated successfully with cement augmentation alone without further spine stabilization have been reported. To our knowledge, there is no report of an elderly female treated with cement augmentation alone for a C2 plasmacytoma without subsequent cervical instability in the orthopedic literature. This case demonstrates the possibility of favorable outcomes in elderly patients with plasmacytoma treated with cement augmentation alone, as opposed to cement augmentation with further spinal stabilization, which may pose additional morbidity and mortality.

## 2. Case

An 83-year-old woman presented to the emergency department with a one-day nocturnal history of cervicalgia, dysphagia, and right scapular pain. She also complained of limited range of motion of the neck, and cervicalgia exacerbated by movement. She did not admit to any history of falls, numbness or tingling, or systemic complaints. Physical exam yielded tenderness to palpation in the cervical region. No sensory or motor neurological deficits were noted. Vitals signs, metabolic panel, and complete blood count were within normal limits. In the emergency department, CT of the cervical spine showed a large C2 lytic lesion with cortical breakthrough in the inferior plate, as well as anteriorly. There was no vertebral height loss or collapse, and the C1-C2 articulations were intact.

Subsequent serum and urine protein electrophoresis indicated possible monoclonal gammopathy with serum immunofixation. Bence-Jones proteins were present. Bone survey indicated significant bone demineralization and arthritic changes with repeat demonstration of the lytic lesion at C2. No lesions of the right scapula or proximal ribs were noted.

It was decided, based on the patient's age, poor bone quality, and multiple comorbidities, a C1–C3 fusion would pose excess risk to the patient. Thus, it was decided to perform intralesional curettage and cement augmentation. The patient was informed that if cement augmentation were to fail, a fusion could still be necessary later on.

**Figure 1:**
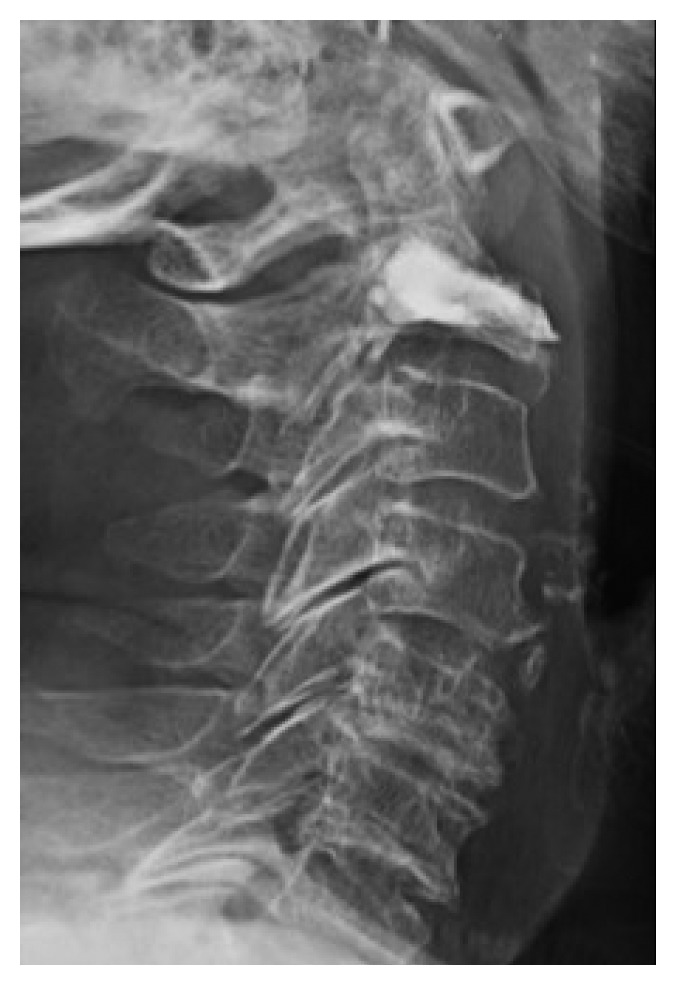
Lateral extended view showing bone filter and methylmethacrylate filled into C2 curettage.

**Figure 2:**
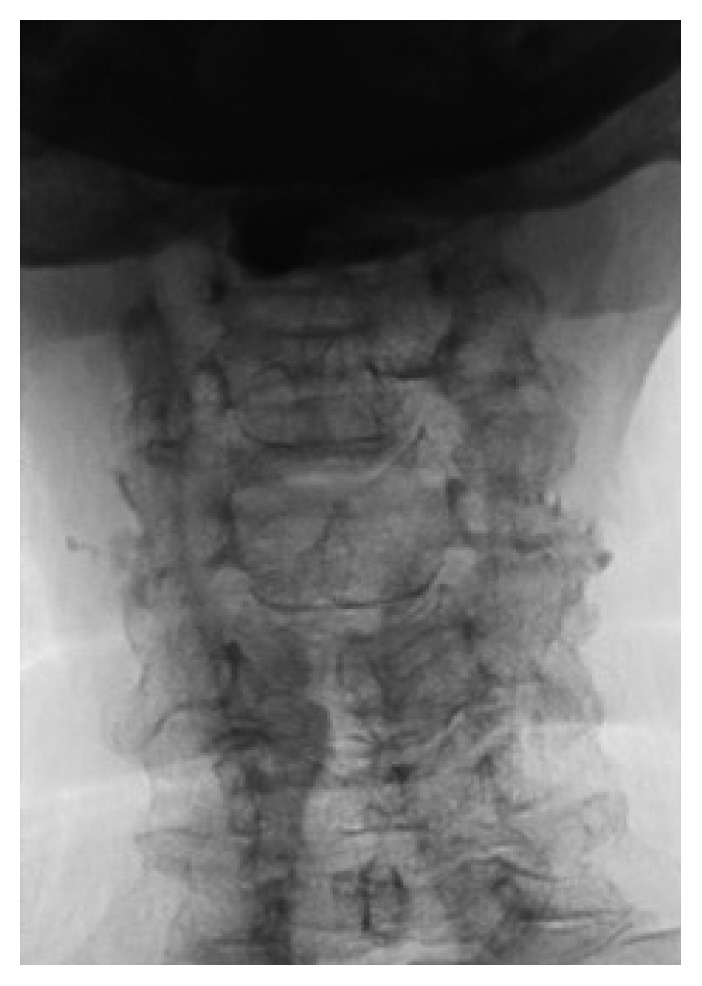
AP view with black/white reversal.

Intraoperatively, an anterior cervical approach was used. Then a small cortical window was made on the inferior aspect of the C2 vertebra. The tumor was suctioned and curetted and filled with iohexol contrast agent to confirm the absence of posterior leakage. Next, 2.5 mL of methyl methacrylate and bone filler were infiltrated into the vertebra. Intraoperative and postoperative AP and lateral X-rays confirmed no cement had extravagated posteriorly (Figures 1 and 2). Final pathologic diagnosis of the lesion and a bone marrow core biopsy revealed plasma cell myeloma with kappa restriction.

At 9 months post-op, the patient's neck and scapular pain was 100% improved and fully resolved. She progressed well without any evidence of complication or loosening around cement augmentation or cervical instability. At that time, she was to follow up on a PRN basis only and continue home exercises including isometrics and ROM.

## 3. Discussion

SBP in the CCJ is a rare entity that typically occurs in younger males, and is responsive to radiation. When surgical intervention is needed, cement augmentation with further stabilization is routinely recommended to avoid instability of the CCJ. Those treated with cement augmentation alone have previously been reported to develop cervical instability and need future stabilization and fusion procedure [6].

To our knowledge, this is the first case in the spine literature of an elderly female successfully treated with cement augmentation alone for a C2 plasmacytoma without subsequent cervical instability. Our case is unique as SBP was treated with cement augmentation alone in an elderly woman with favorable outcome. At 9-month follow-up, she had no signs of cervical instability, and her cervicalgia was completely resolved. This case illustrates how cement augmentation alone may serve to limit morbidity and mortality in elderly individuals when compared to combined cement augmentation with fusion. We will briefly review treatment modalities described for SBP of the spine cited in the literature.

In a systemic review and proposed treatment algorithm, Ahmadi et al. describe a series of four patients with a mean age of 58 with cervical plasma cell neoplasms. One lesion was present at C1, while the others were found at C2. Two patients received vertebroplasty and radiotherapy as primary management, and the others received occipitocervical stabilization (OCS). The patients who received vertebroplasty alone developed secondary instability of the CCJ, requiring OCS at 5 and 12 months, while patients who underwent OCS alone did not require any further surgical intervention. Given these results and a review of the literature, OCS for SBP in the CCJ was stated to be the favored surgical treatment option [6].

Lourbopoulos et al. described an unusual presentation of plasmacytoma presenting as a C4–7 epidural mass causing progressive upper and lower extremity weakness and numbness in a 45-year-old male. The patient's ascending paralysis initially led to a misdiagnosis of Guillain-Barre syndrome, which was then correctly diagnosed as a cervical epidural plasmacytoma. He was then treated with C4–7 laminectomy alone. At 9- and 13-month follow-up visits, he was in complete remission, was able to ambulate with unilateral assistance, and continued to gain strength [7].

Three cases of transoral resection of axial lesions augmented by intraoperative MRI have also been reported. Each case demonstrated different lesions involving the CCJ. Intraoperative MRI was used to determine adequacy of decompression. In one case, intraoperative MRI led to a change from the planned surgical approach. Intraoperative MRI did not adversely affect operating times or neurosurgical technique including instrumentation requirements [8].

In 2004, Çolak performed a retrospective review of eight cases with C2 vertebral body neoplastic lesions to determine the effectiveness of a two-staged surgery and outcomes. Patients were first decompressed via an anterior approach and then stabilized posteriorly at a later time. It was found that the staged approach increased patient tolerability without increasing morbidity [9].

To our knowledge, this is the first case in the spine literature of an elderly female successfully treated with cement augmentation alone for a C2 plasmacytoma without subsequent cervical instability. At 9-month follow-up, she had no signs of cervical instability, and her cervicalgia was completely resolved. This case illustrates how cement augmentation alone may serve to limit morbidity and mortality in elderly individuals when compared to combined cement augmentation with fusion.

Our case represents another treatment option for solitary lesions within the CCJ. Our patient, an elderly female, demonstrated favorable outcome with cement augmentation and curettage for a C2 plasmacytoma without need for further stabilization or fusion.

## 4. Conclusion

SBP is a rare disease whose surgical treatment entails cement augmentation with further stabilization. Our case demonstrates successful surgical treatment in an elderly female patient with a C2 plasmacytoma utilizing cement augmentation and curettage without need for further stabilization or fusion. This may be a potential definitive surgical treatment option in patients with high morbidity and mortality.
